# Free-aldehyde neutralized and oligohyaluronan loaded bovine pericardium with improved anti-calcification and endothelialization for bioprosthetic heart valves

**DOI:** 10.3389/fbioe.2023.1138972

**Published:** 2023-04-03

**Authors:** Yuhong Liu, Chunyang Chen, Ting Lu, Sixi Liu, Zhongshi Wu, Zhenjie Tang

**Affiliations:** ^1^ Department of Cardiovascular Surgery, The Second Xiangya Hospital of Central South University, Changsha, Hunan, China; ^2^ Engineering Laboratory of Human Province for Cardiovascular Biomaterials, Changsha, Hunan, China

**Keywords:** bioprosthetic heart valves, adipic dihydrazides, oligo-hyaluronan, anti-calcification, endothelialization

## Abstract

The number of patients with valvular heart disease is increasing yearly, and valve replacement is the most effective treatment, during which bioprosthetic heart valves (BHVs) are the most widely used. Commercial BHVs are mainly prepared with glutaraldehyde (Glut) cross-linked bovine pericardial or porcine aortic valves, but the residual free aldehyde groups in these tissues can cause calcification and cytotoxicity. Moreover, insufficient glycosaminoglycans (GAGs) in tissues can further reduce biocompatibility and durability. However, the anti-calcification performance and biocompatibility might be improved by blocking the free aldehyde groups and increasing the GAGs content in Glut-crosslinked tissues. In our study, adipic dihydrazide (ADH) was used to neutralize the residual free aldehyde groups in tissues and provide sites to blind with oligohyaluronan (OHA) to increase the content of GAGs in tissues. The modified bovine pericardium was evaluated for its content of residual aldehyde groups, the amount of OHA loaded, physical/chemical characteristics, biomechanical properties, biocompatibility, and *in vivo* anticalcification assay and endothelialization effects in juvenile Sprague-Dawley rats. The results showed that ADH could completely neutralize the free aldehyde groups in the Glut-crosslinked bovine pericardium, the amount of OHA loaded increased and the cytotoxicity was reduced. Moreover, the *in vivo* results also showed that the level of calcification and inflammatory response in the modified pericardial tissue was significantly reduced in a rat subcutaneous implantation model, and the results from the rat abdominal aorta vascular patch repair model further demonstrated the improved capability of the modified pericardial tissues for endothelialization. Furthermore, more α-SMA^+^ smooth muscle cells and fewer CD68^+^ macrophages infiltrated in the neointima of the modified pericardial patch. In summary, blocking free-aldehydes and loading OHA improved the anti-calcification, anti-inflammation and endothelialization properties of Glut-crosslinked BHVs and in particularly, this modified strategy may be a promising candidate for the next-generation of BHVs.

## 1 Introduction

Valvular heart disease (VHD) seriously threatens human health worldwide, especially in elderly individuals, and prosthetic valve replacement has become the most effective treatment strategy for VHD due to the lack of effective drugs for prevention and treatment (Yan et al., 2010). With the increase in the aging population population increases, the number of VHD patients requiring valve replacement is increasing every year (Fioretta et al., 2018). Bioprosthetic heart valves (BHVs) are the predominant treatment option for VHD (Xue et al., 2022). Due to the significant advantages of BHVs in terms of hemodynamics and hemocompatibility, in addition to elderly patients, younger patients and active patients are also prefer BHVs (Lim, 2018; Meuris et al., 2018; Catterall et al., 2020). In addition, BHVs are widely used in transcatheter heart valve replacement, which further expands their clinical applications (Khan et al., 2020).

Currently, clinically available BHVs are mainly prepared from glutaraldehyde (Glut) fixed xenograft biomaterials such as bovine pericardium or porcine aortic valve (Head et al., 2017). Although Glut-crosslinked valves have good biomechanical properties, they have been shown to induce several negative effects that result in poor durability, including the presence of residual free aldehyde groups, toxicity, calcification, induction of the inflammatory response and poor endothelialization (Gong et al., 1991; Levy, 1994; Schoen, 2008). The persistently unbound residual aldehyde groups in BHVs might directly bind with calcium ions to form calcification sites (Gong et al., 1991; Levy, 1994), and then can also induce cell lysis around the xenografts, leading to the release of cellular debris and phospholipid cell membrane debris (Thiene and Valente, 2011), and inhibiting endothelialization (Hoffman et al., 1992). Serum calcium reacts with residual phosphorus in cell debris to generate calcium phosphate apatite crystals, which allows the formation of some potential calcification sites (Pettenazzo et al., 2008; Meng et al., 2021).

The neutralization of free aldehyde groups has been shown to reduce tissue calcification and toxicity, such as with amino acids, sodium borohydride, citric acid, and α-amino oleic acid (Kim et al., 2011; Lim et al., 2015; Jiang et al., 2022). Adipic dihydrazide (ADH) is homobifunctional cross-linking reagents specific for aldehydes that generate relatively stable hydrazone linkages (Chen et al., 2018). ADH reacts with free aldehyde groups in the pericardium and increases the number of primary amine groups in tissues. The heart valve matrix is rich in glycosaminoglycans (GAGs) and proteoglycans (approximately 30%), mainly hyaluronan (HA), which forms the hydrophilic matrix of the valve and maintains valve homeostasis (Gupta et al., 2009; Lehmann et al., 2009). GAGs are also critical in regulating the movement of heart valves, providing compressive strength and allowing shear between the layers during flexure (Simionescu et al., 2003). In addition, HA, composed of repeating disaccharides of N-acetyl-glucosamine and glucuronic acid, can bind to cell surface receptors to influence a variety of biological processes, including angiogenesis, wound healing and cell migration and proliferation (Knudson and Knudson, 1993; Rooney et al., 1993). Polymer size appears to endow HA fragments with specific functions. Large HA polymers are antiangiogenic and immunosuppressive and hinder differentiation, possibly by inhibiting cell-to-cell interactions or ligand access to cell surface receptors (Stern et al., 2006; Rayahin et al., 2015). Smaller fragments function through immunostimulation, angiogenesis, cell adhesion and differentiation. In addition, HA has a space filling function that competitively prevents ion binding (Stern et al., 2006). Studies have shown that the 5–10 kD oligohyaluronan (OHA) fragment can promote angiogenesis and promote endothelial cell migration and mitosis (Slevin et al., 2002). Taken together, these findings suggest that reducing the free aldehyde groups in Glut-crosslinked bovine pericardial valves while increasing the amount of OHA is a promising strategy to facilitate healthy tissue formation.

We hypothesized that neutralizing the residual free aldehyde groups in the Glut-crosslinked bovine pericardium by ADH and increasing the amount of HA loaded in the tissue would improve the biocompatibility, anti-calcification and endothelialization effects of the BHVs. In this study, the effects of blocking the free aldehyde groups on the Glut-crosslinked bovine pericardium loaded with OHA were assessed, and the content of free aldehyde groups and the amount of OHA loaded, biomechanical properties, physical/chemical characteristics, *in vitro* cytotoxicity, proliferation effects and hemocompatibility were evaluated. Moreover, calcification and endothelialization were further explored in a rat subcutaneous implantation model and abdominal aorta vascular patch repair model. To our knowledge, this is the first demonstration that OHA can accelerate the endothelialization of valves and that ADH can completely neutralize free aldehyde groups.

## 2 Methods and materials

**FIGURE 1:**
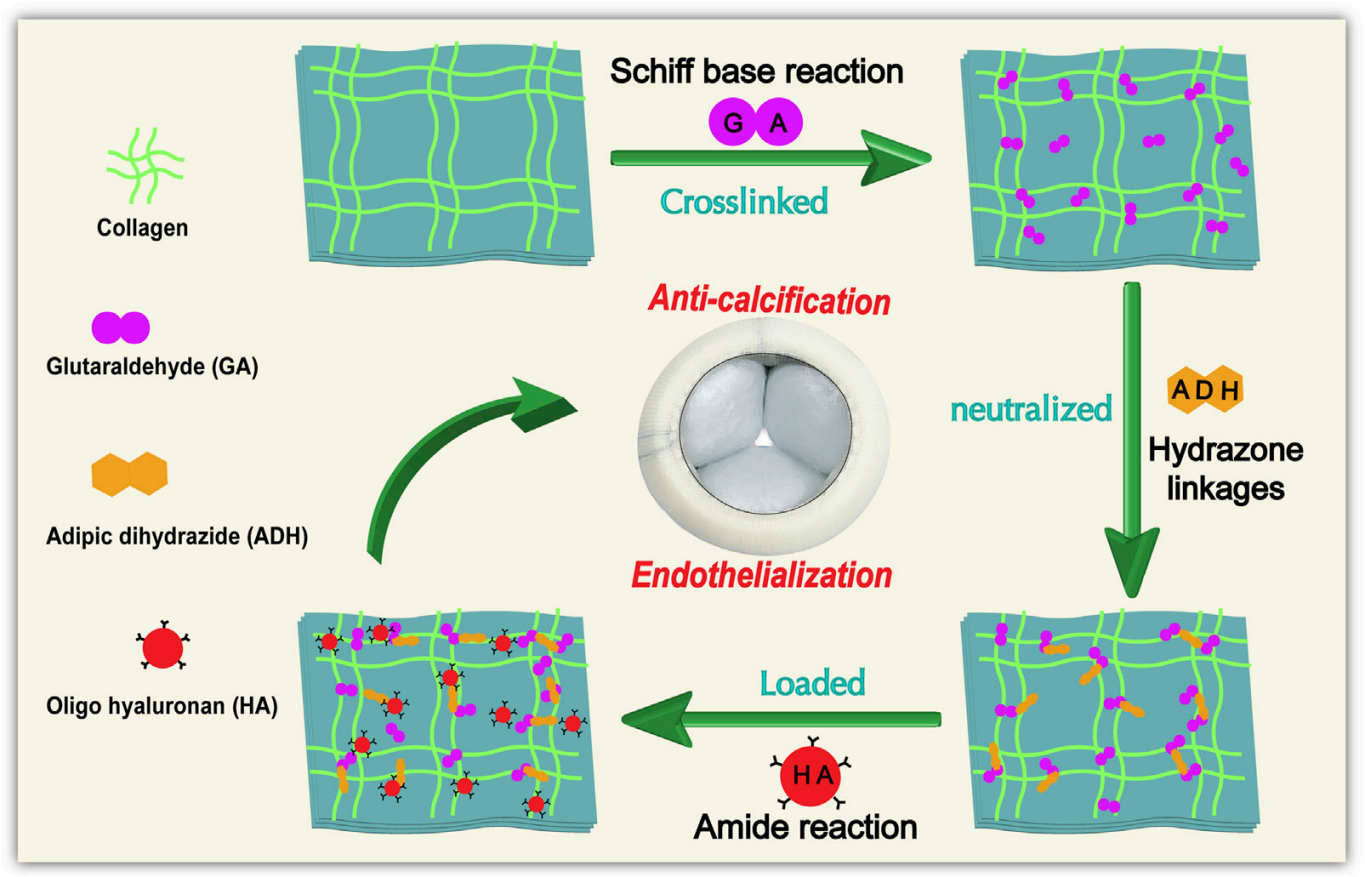
General procedure and reaction principle for the treatment of Glut-crosslinked bovine pericardium modification.

Yellow cattle pericardium with a warm ischemia time of less than 30 min was collected from a local slaughterhouse. The remaining fat and tissue were removed, and the pericardium was then cleaned with phosphate buffered saline (PBS). The pericardium was cut into leaflets, each with a size of 10 cm × 10 cm, and stored in PBS with 1% penicillin/streptomycin at 4°C. Thereafter, the leaflets were further processed as indicated in our scheme (Figure 1).

### 2.1 Glut crosslinking and free aldehyde blocking

The pericardial leaflets were immersed in a buffered saline solution (pH 7.4) with 0.6% Glut (Aladdin, G105905, China) and 50 mM HEPES (Sigma, H3375, United States) at ambient temperature. The solution was changed to a 0.2% GLUT solution after 24 h and maintained for an additional 6 days at ambient temperature. After crosslinking, the samples were rinsed with PBS and store in PBS with 1% penicillin/streptomycin at 4°C. (GA samples).

For blocking the free aldehyde groups, different concentrations of ADH solution (25, 50, and 100 mM) were provided by dissolving the corresponding content of ADH in a 300 mL of 50 mM HEPES buffer solution (pH = 5.0). Glut-crosslinked leaflets were then immersed in 300 mL of the above solution for 24 h at ambient temperature with gentle shaking. After the reaction was complete, the samples were rinsed thoroughly with PBS and placed in short-term storage at 4°C for the subsequent processing. (ADH samples).

### 2.2 Immobilization of oligo-hyaluronan onto pericardium

The ADH samples and GA samples were covalently modified with oligohyaluronan by carbodiimide chemistry. Briefly, the OHA (1.5 g/mL) were added to 300 mL of MES buffer (pH = 5.5) with N-hydroxysuccinimide (NHS, 2 mg/mL) and 1-(3-dimethylaminopropyl)-3-ethyl carbodiimide hydrochloride (EDC·HCl, 5 mg/mL). Importantly, the pH value of the reaction system was maintained at approximately 5.5 for 30 min to activate the carboxyl group. Then the free aldehyde-blocked pericardium was immersed in the above solution, the pH value was adjusted to approximately 7.2, where it remained for the entire 24 h of reaction under constant stirring at 37°C. After the reaction was complete, the samples were rinsed thoroughly with PBS and placed in short-term storage at 4°C. (OHA samples) All the modified leaflets were sterilized by irradiation (radiation dose: 25 kGy).

### 2.3 Free aldehyde group quantification and visualization

For visual evaluation, pericardial patches from each group (*n* = 5) were incubated in Schiff reagent (Phygene, PH0643, China), which can react with aldehyde groups to form a purple-red color, for 30 min (Meuris et al., 2018). The free aldehyde content was quantified with an acetaldehyde dehydrogenase (ALDH)-based assay using 2 U/mL ALDH (Sigma‒Aldrich, 9028, United States) in 100 mM Tris HCl buffer (pH 8.5) with oxidized nicotinamide adenine dinucleotide (NAD+; 0.8 mM, Sigma, NAD100, United States) as a cofactor. A standard curve of nicotinamide adenine dinucleotide (NADH) and Glut was used for the quantification (linear 0–0.5 mM range; *n* = 5). The free aldehyde group content within the unit area of each group was measured. NADH quantification was determined by measuring the absorbance at 340 nm (Lopez-Moya et al., 2018).

### 2.4 Fixation index (FI) of the three groups of samples

The fixation index (FI), which reveals the ratio of free amino groups groups in a materials, was evaluated by using the ninhydrin (NHN) assay (Sung et al., 1996). The free amino content in the sample is positively correlated with the absorbance intensity of the chromogenic substances. Briefly, the three groups of samples were lyophilized for 24 h and then weighed. Specimens of the same weight were then added to 1% NHN solution (Aladdin, N105629, China) and heated at 100°C for 20 min. Then, each group of samples (*n* = 6) was removed and cooled for 20 min. Finally, the absorbance of the obtained solutions was measured by a microplate reader (Thermo, United States) at 570 nm. In addition, calibration curves were constructed with glycine solutions of various known concentrations. The FI was calculated by the following equation: FI (%) = (control-reactive amine-sample-reactive amine)/control-reactive amine × 100%.

### 2.5 Assay of OHA content

To determine the OHA content in the samples (GA/ADH/GA-HA/OHA), the Purple-Jelley HA assay (Biocolor Ltd.) was used (Fede et al., 2018). Briefly, samples were digested in 50 mM Tris–HCl (pH 7.6) containing proteinase K (Sigma Aldrich) overnight at 55°C. All the processes followed the protocol of the kit. The absorbance value at 650 nm was measured with a microplate reader (Thermo, United States).

### 2.6 Histological characterization

To observe the histological morphology of the pericardium, the hematoxylin and eosin (HE, Servicebio, G1005, China) staining, Masson’s trichrome (Servicebio, G1006, China) staining, and Alcian blue staining (Servicebio, G1027, China) were performed. The native pericardium (NA), Glut cross-linked pericardium (GA), free aldehyde blocked pericardium (ADH) and OHA loaded pericardium (OHA) samples were fixed with paraformaldehyde, dehydrated in graded alcohol concentrations, and then embedded in paraffin and sectioned (5 μm). The samples were finally observed using an optical microscope (Nikon, NIS-eLEMENTS d).

### 2.7 Scanning electron microscopy and contact angle test

For scanning electron microscopy (SEM), after fixation, the samples were rinsed with PBS and dehydrated in a graded ethanol series (30%, 50%, 70%, 90%, and 100%) and tert-butanol, followed by freeze-drying. The surfaces of the samples were sputter coated with 20 nm gold for observation with an FEI Nova Nano-SEM (FEI electronic optics B. V, Czech Republic).

The hydrophilicity of the samples was determined with a water contact angle test. In each group, pericardial samples (0.8 cm × 0.8 cm) were lyophilized (*n* = 6) and analyzed on an optical contact angle measurement instrument (SDC-200s, Sheng Ding, China).

### 2.8 Biomechanical properties

An INSTRON instrument was used for the uniaxial tensile testing. Sample from the three groups were cut into dog-bone shaped pieces. Before the test, and the average thickness was calculated by measuring the thicknesses of four randomly selected locations on each sample. The pericardium was subjected to a 200 N sensor and a tensile rate of 10 mm/min until failure. The tensile modulus of the linear region determined from the stress-strain curve within the strain of 0%–10%. In addition, the ultimate tensile stress, ultimate tensile strain, and representative stress-strain curves were also obtained.

### 2.9 *In vitro* resistance to enzymatic hydrolysis assay

To test the resistance of the GA/ADH/OHA materials to collagenase, the specimens were cut into leaflets of 1 cm × 1 cm (*n* = 6), rinsed with deionized water, frozen, lyophilized, and weighed. The samples were digested for 24 h at 37°C in 1.0 mL of 150 U/mL collagenase in 50 mM TES. All digested samples were then washed with deionized water, frozen, and lyophilized, and weighed. The percent weight loss was calculated.

### 2.10 The Fourier transform infrared spectroscopy and thermal analysis

The Fourier transform infrared (FTIR) spectra were collected with an FTIR spectrometer equipped with a photomultiplier detector (iS-50, Thermo Scientific Nicolet). The samples were ground with liquid nitrogen, lyophilized, and pressed into pellets. Spectra were obtained by recording 48 scans between 4,000 and 500 cm^−1^ with a resolution of 4 cm^−1^.

Thermal analysis was conducted by differential scanning calorimetry (DSC, 204F1, NETZSCH, Germany) and thermogravimetric analysis (TGA, TGA209F1, NETZSCH, Germany) (Shamis et al., 2009). The vacuum chamber was operated in accordance with the manufacturer’s instructions to ensure that the samples were free of moisture. Briefly, freeze-dried (5–10 mg) samples were equilibrated at 30°C, then assessed by DSC in the range of 30°C–150°C under a N_2_ atmosphere with a heating rate of 10°C/min and by TGA in the range of 30°C–1,000°C under a N_2_ atmosphere with a heating rate of 20°C/min. The thermal denaturation temperature and thermogravimetric data from the first heating curve of the samples were recorded.

### 2.11 Cytocompatibility assay

Radiation-sterilized GA, ADH and HA samples (1 cm × 1 cm) were washed with sterile PBS three times and immersed in high glucose Dulbecco’s Modified Eagle Medium with 10% fetal bovine serum (DMEM/10% FBS, Gibco, United States) cell culture medium for 24 h. Then the sheets were placed in a 24 well plate, and human umbilical vein endothelial cell line (HUVECs, line EAhy926) were seeded on the sheets at a density of 30,000 cells/well. After 5 days of incubation, the 100 µL of carboxyl-2′,7′-dichlorofluorescein diacetate live cell fluorescent dye solution was added to the well plate and incubated for 30 min, and then the morphology and proliferation of HUVECs on the sheets were observed by the fluorescence microscopy (Nikon, NIS eELEMENTS d).

For the cytotoxicity assay, pericardial sheets (1 cm^2^ × 1 cm^2^) were cut into pieces, immersed in DMEM/10% FBS culture medium at a density of 2.5 mL/cm^2^, cultured at 37°C for 24 h, and the leach liquor was collected. HUVECs were cultured in DMEM/10% FBS. A total of 5,000 cells were seeded in 96-well plates (*n* = 6), and 200 μL of medium containing DMEM/10% FBS and leach liquor diluted 1:1 was added. The cells were cultured at 37°C under 5% carbon dioxide for 1, 3 and 5 cm^2^ days. The negative control sample was prepared with DMEM/10% FBS only. Cell growth was evaluated by measuring the mitochondrial metabolism (MTT) and optical density (OD) at 570 nm with a microplate reader (Thermo Scientific, Multiskan Sky). The relative growth rate (RGR) was used as a measure of cytotoxicity in each group, with RGR = (mean OD for each group)/(mean OD of the negative control) × 100%.

### 2.12 Platelet adhesion and hemolysis

A platelet adhesion assay was used to evaluate platelet behavior in each sample. Fresh whole blood from healthy individuals was collected and anticoagulated with sodium citrate plus sodium citrate (1:9). Platelet-rich plasma (PRP) was prepared by centrifuging the samples at 1,500 rpm for 15 min at 4°C. Pericardial sheets (1 cm × 1 cm) were placed into 48-well plates and immersed in PBS at 37°C for 2 h, after which the solution was replaced with 400 μL of fresh PRP and for 1 h of incubation at 37°C. Then, the PRP was discarded, and the samples were washed with PBS three times. For SEM observations, the pericardial sheets were fixed with 3% Glut for 30 min, and then gradient dehydration and freeze-drying were performed as described previously. In addition, for lactate dehydrogenase (LDH) ELISAs, pericardial sheets were lysed with 0.5% Triton X-100 (v/v) at room temperature for 30 min (*n* = 6). The supernatant was collected, and the content of LDH was measured with an LDH test kit (China Yancheng A020).

For the hemolysis assay, the above sedimented red blood cells (RBCs) were washed with normal saline (NS) three times. After each washing step, the solution was centrifuged at 3,500 rpm for 5 min. The RBC concentrate was diluted with NS to a final concentration of 5 vol%. The samples were soaked in 1 mL of 5% RBC suspension and incubated for 1 h at 37°C (*n* = 6). In addition, 300 μL of NS and 300 μL of 1% SDS were added to the RBC suspension as the negative control (0% lysis) and positive control (82% lysis), respectively. After centrifugation at 3,000 rpm for 10 min, the absorbance of the supernatant at 540 nm was measured with a microplate reader. To calculate the hemolysis ratio, the following equation was used.

$$Hemolysis\;ratio=\left(sample-negative\;control\right)/\left(positive\;control-negative\;control\right)\times 100\%$$

### 2.13 *In vivo* rat aorta patch repair model

All animal experiments were approved by the Institutional Animal Care and Use Committee (IACUC), The Second Xiangya Hospital, Central South University, China. All operations were conducted while keeping with the Guide for Care and Use of Laboratory Animals. Endothelial cell coverage in three groups (GA, ADH and OHA) was evaluated in a rat infrarenal abdominal aorta patch repair models. The rats were anesthetized with 30 mg/kg sodium pentobarbital, the patch of sterile samples were implanted in male juvenile Sprague Dawley rats’ abdominal aorta (SD, *n* = 5). Briefly, a portion of the infrarenal abdominal aortic wall (2 mm × 10 mm) was excised from the isolated abdominal artery, and a pericardial patch (3 mm × 10 mm) was sutured on the abdominal aortic wall with 7-0 prolene sutures. All samples were retrieved 4 weeks after implantation. Then, each sample was fixed with paraformaldehyde and saved for histological analysis. The fixed implants from each group of samples were gradient-dehydrated, then embedded in paraffin and sectioned (5 μm) for light microscopy analysis. The samples were analyzed histologically using the HE staining. In addition, the endothelial cells, smooth muscle cells (SMCs) and macrophage cells in the neointima were identified by immunofluorescence (IF) with CD31 antibody (1:200, Abcam, ab9498, United Kingdom), α-SMA antibody (1:800, GeneTex-60466, United States) and CD68 antibody (1:200, GeneTex-41868, United States). Briefly, the sections were deparaffinized, rehydrated and processed for antigen retrieval. Then the sections were incubated with primary antibodies at 4°C overnight followed by incubation with secondary antibodies, nuclei were counterstained with 4′,6-diamidino-2-phenylindole (DAPI), tissue autofluorescence was quenched, and images were acquired with a fluorescence microscope.

### 2.14 *In vivo* rat subcutaneous implantation

Calcification in the different groups (GA/ADH/OHA) was evaluated in subcutaneous implantation rat models. Sterile samples (1 cm^2^ × 1 cm^2^) were implanted subcutaneously in male juvenile SD rats (*n* = 8) anesthetized with pentobarbital sodium (30 mg/kg). A longitudinal incision was made on the rat’s back to prepare a subcutaneous pocket for the pericardial samples. A 2-0 Mersilk suture was used to seal the incision. For calcification evaluation, the samples were retrieved 60 days after implantation. Then, a portion of the sample was fixed with paraformaldehyde and saved for histological analysis. The fixed implants from each group of samples were gradient dehydrated, then embedded in paraffin and sectioned (5 μm) for light microscopy analysis. H/E stain and Alizarin red staining were used to visualize and assess the histological morphology and the distribution of the calcium deposition in the implanted samples from each group. Furthermore, the calcium contents were analyzed by inductively coupled plasma-optical emission spectrometer (ICP-OES, Thermo Fisher, United States) (Qi et al., 2022). Briefly, the fibrous capsule tissue in the specimen was carefully removed with forceps. The obtained tissues were lyophilized, weighed, and dissolved with 6 N hydrochloric acid at 96°C for 24 h. After the above solution was filtered, it was diluted 1:15 with deionized water and then measured. The content of calcium was normalized to the dry weight of the implanted samples.

Immunocompatibility in the different groups (NA/GA/ADH/OHA) was evaluated in subcutaneous implantation rat models (*n* = 5). The Immunohistochemistry (IHC) staining for CD68 and CD3 was performed on specimens implanted at 2 weeks *in vivo*, the sections (5 μm) were deparaffinized, rehydrated and processed for antigen retrieval (Heat induced antigen retrieval was performed in 10 mM Tris with 1 mM EDTA buffer with high temperature and pressure for 3 min). Then the sections were incubated with primary antibodies at 4°C overnight. The CD68 antibody (1:200, GeneTex-41868, United States) was used to label the macrophage cells, and the CD3 antibody (1:700; Servicebio, GB11014, China) was used to label the T cells. At least five fields were counted for the number of inflammatory cells in each specimen.

### 2.15 Statistical analysis

The results of the quantitative studies were expressed as the mean ± standard error of the mean (SEM). The Shapiro-Wilk normality test was used to define whether data were normally distributed. One-way analysis of variance (ANOVA) was conducted to determine differences between three groups for continuous variables with normal distribution. The Kruskal–Wallis test with Tukey’s *post hoc* test was conducted to evaluate the differences among non-normally distributed data. All the statistical analyses were performed with GraphPad Prism 8.4.0 (GraphPad Software, United States). A *p*-value <0.05 was considered to be statistically significant.

## 3 Results

### 3.1 ADH significantly reduced the residual free aldehyde groups in Glut cross-linked bovine pericardium

**FIGURE 2:**
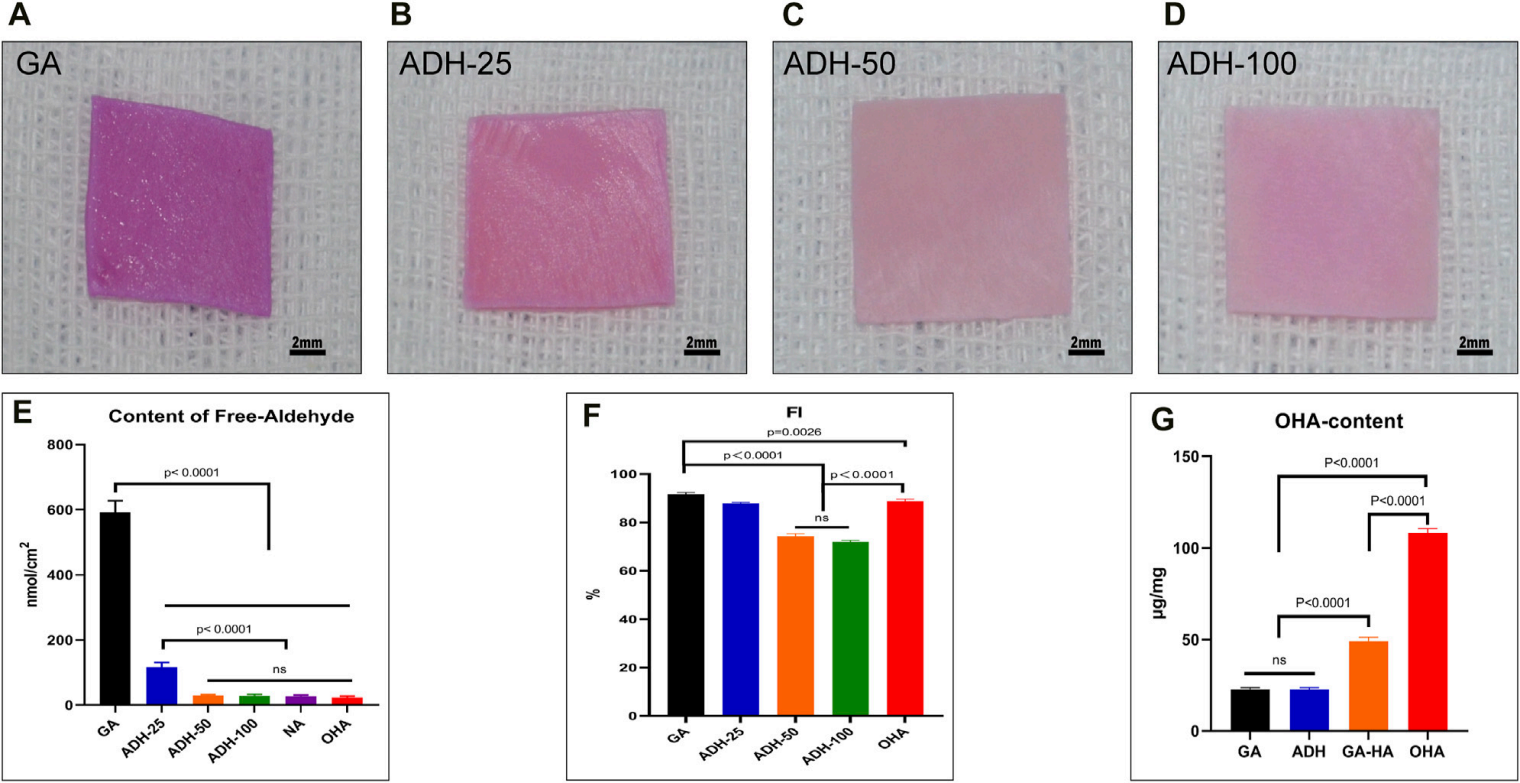
Qualitative and quantitative analysis of free aldehyde groups in pericardial tissues. The images show the staining results of Schiff base reagents, and purple-red represents the residual aldehyde groups **(A–D)**. The results of quantitative analysis showed that the residual aldehyde groups in the GA group were significantly higher than those in the ADH and OHA groups **(E)**. Quantitative analysis of free amino groups in tissues **(F)**. Qualitative analysis of OHA content in tissues **(G)**.

Fresh pericardial tissue is often fixed with Glut, which chemically combines aldehydes with free amine groups with the proteins (mainly collagen) on tissue to obtain GA samples (Zhai et al., 2006). To reduce the free aldehyde groups in the tissues, ADH was employed to combine with the reactive aldehyde groups in tissues to generate relatively stable hydrazone linkages. As shown in the results of Schiff reagent staining results, the GA group showed obvious purplish red staining (Figure 2A) suggesting the presence of free aldehyde groups. In addition, the 25 mmol/L ADH group still showed a local purplish red, which indicated that there were still some residual free aldehyde groups in this tissue (Figure 2B). In the other two ADH groups (50 mmol/L and 100 mmol/L) there was only light red staining in the bovine pericardium (Figures 2C, D). Furthermore, an ALDH-based assay was utilized to evaluate the contents of the free aldehyde groups in the tissues from each group (Figure 2E). The results showed that there were 591.7 ± 14.5 nmol/cm^2^ residual free aldehyde group in the GA group, and 116.5 ± 5.9 nmol/cm^2^ in the 25 mmol/L-ADH group. In the 50 mmol/L-ADH (29.6 ± 1.2 nmol/cm^2^), 100 mmol/L-ADH (28.9 ± 1.6 nmol/cm^2^), OHA (23.6 ± 1.5 nmol/cm^2^) and NA (27.3 ± 1.4 nmol/cm^2^) groups, the contents of free aldehyde groups were significantly lower than that in the GA group.

### 3.2 ADH increased both the content of reactive amino group and the loading of OHA

To investigate whether there were reactive amino groups in the tissues after ADH treatment, a NHN assay was performed (Figure 2F). The FI was 91.7% ± 0.6% in the GA group, 87.9% ± 0.4% in the 25 mmol/L ADH group, 74.3% ± 1% in the 50 mmol/L ADH group, and 71.9% ± 0.6% in the 100 mmol/L ADH group. These results showed that ADH treatment significantly decreased the FI of Glut-crosslinked pericardium, indicating that there were still reactive amino groups present after neutralization of the residual aldehyde groups in the tissues that could be used as reactive groups for OHA loading. Considering the above results, the 50 mmol/L ADH group was selected for subsequent experiments. In addition, after OHA was loaded by carbodiimide chemistry, the FI of the OHA group (88.7% ± 0.9%) was significantly higher than that of the ADH group. Moreover, the loading of OHA content was evaluated (Figure 2G), and the results showed that the loading content in the OHA group (108.7 ± 2.4 μg/mg) was significantly higher than that in the GA-HA group (48.9 ± 2.2 μg/mg), which indicated that after aldehyde neutralization, the increase in the reactive amino groups in the tissues could react with OHA to improve the loading capacity. The content of HA in the GA and ADH groups was significantly lower than that in the OHA group.

### 3.3 Histological and ultrastructural characterization of the modified pericardium

**FIGURE 3:**
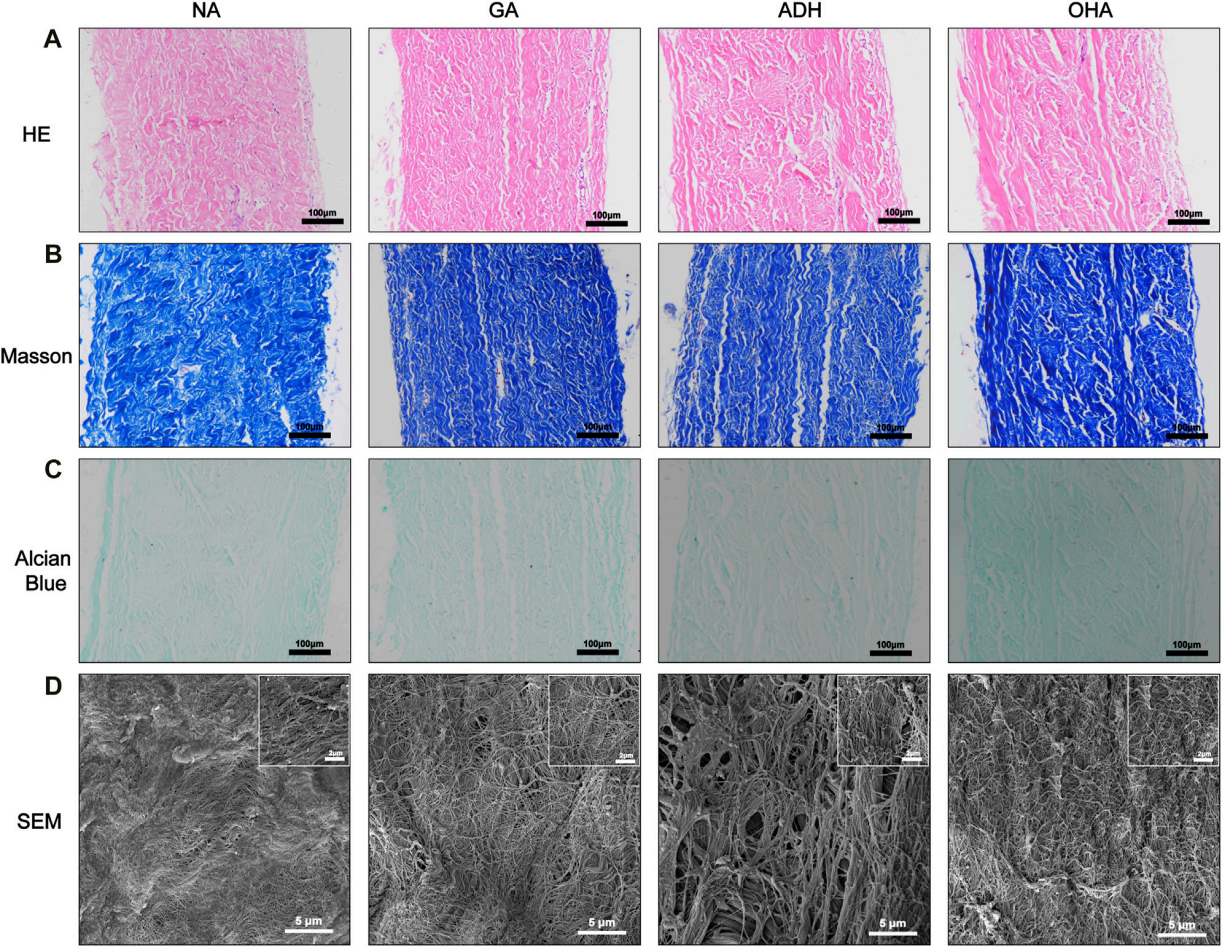
Histological staining and SEM images of the three groups. The images show HE staining **(A)**, Masson’s trichrome staining **(B)**, Alcian blue staining **(C)** and SEM **(D)**.

**FIGURE 4:**
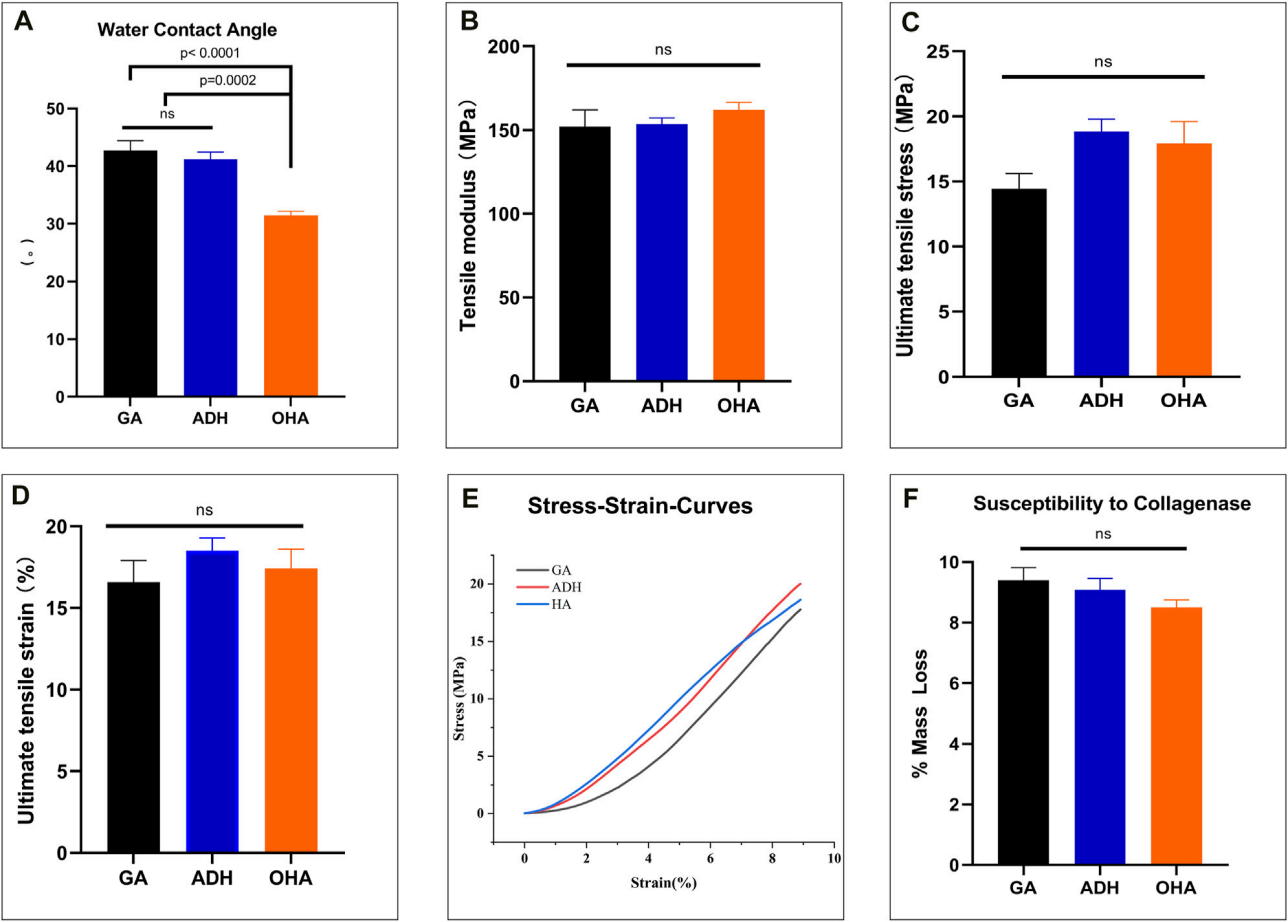
The water contact angle, biomechanical properties and susceptibility to collagenase of the three groups. The water contact angle of the leaflets decreased significantly in the OHA group compared with those of the GA and ADH groups **(A)**. There was significant improvement in the NPTD group compared with the GA group **(D)**. The tensile modulus of the linear region **(B)**, ultimate tensile stress **(C)** and ultimate tensile strain **(D)** among the three groups were no significant difference. The representative stress‒strain curves of each group are presented **(E)**. There was no difference in the relative mass loss among the three groups **(F)**.

To observe the histological characteristics of the samples in the NA, GA, ADH and OHA groups, the samples were analyzed by histological staining, SEM, and water contact angle measurements were utilized. The structure of the extracellular matrix (ECM) was visualized by HE (Figure 3A), Masson’s trichrome (Figure 3B) and Alcian blue staining (Figure 3C). After crosslinking, the structure of the fibers became condensed in the GA group, and similar structures were observed in the ADH and OHA groups. After Alcian blue staining, a deeper blue color was visible in the tissues of the OHA group, which indicated that more HA was contained in these tissues compared with the tissues in the GA and ADH groups. The SEM results further revealed that there was no obvious difference among the three groups in terms of microstructure (Figure 3D), which indicated that modification by ADH neutralization and OHA loading had only a small influence on the morphology of the GA group of samples. To investigate the changes in pericardial hydrophilicity after modification, the water contact angles were measured, and the data showed that the water contact angle was approximately 40° in the GA and ADH groups and approximately 30° in the OHA group (Figure 4A). Thus, the water contact angle in the OHA group was significantly lower than that in the GA and ADH groups, indicating better hydrophilicity of the OHA samples.

### 3.4 Biomechanical properties and collagen stability

To evaluate the biomechanical properties and collagen stability of the three types of pericardium, a uniaxial tensile test and collagenase digestion assay were performed. The results of the uniaxial tensile mechanical test showed an increases in the tensile modulus of the linear region, ultimate tensile stress and ultimate tensile strain in the ADH and OHA groups compared with the GA group, but there was no significant difference among the three groups (Figures 4B–D). In addition, the representative stress‒strain curves of each group were presented in Figure 4E. Collagenase hydrolysis treatment was used to simulate the degradation behavior *in vivo*. The results showed that the mass loss in the OHA group was the minimal after 24 h of enzymatic hydrolysis (8.5% ± 0.3%), while those in the ADH group and GA groups were 9.1% ± 0.4% and 9.4% ± 0.4%, respectively, but no significant differences were observed among the three groups (Figure 4F).

### 3.5 FTIR spectroscopy and thermal analysis

**FIGURE 5:**
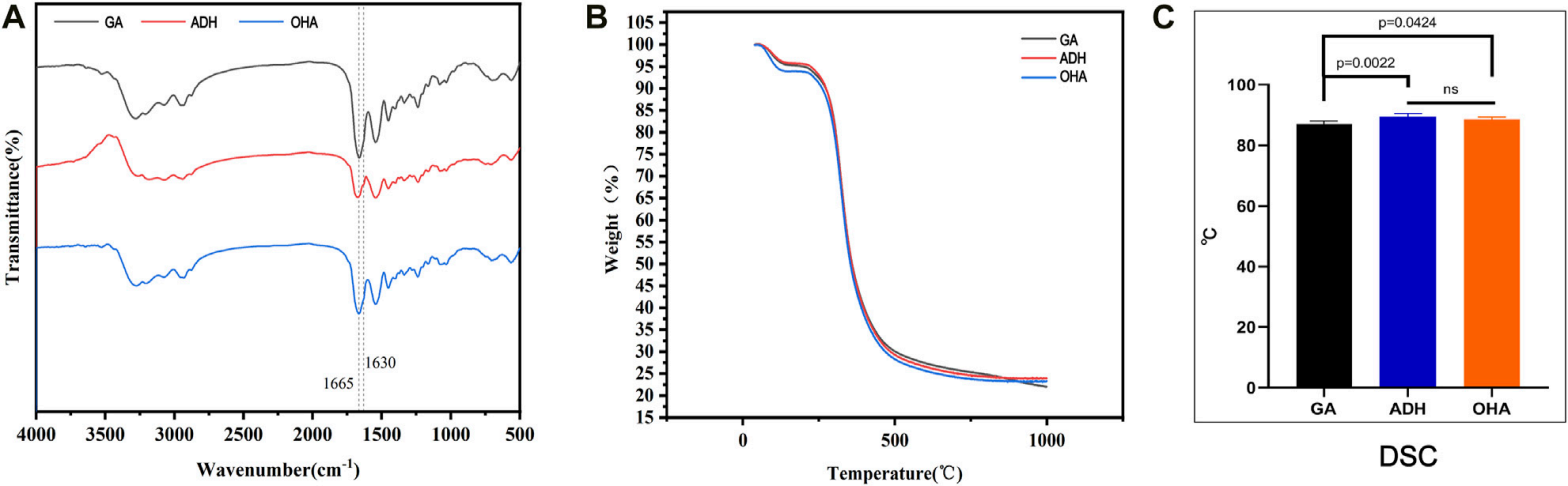
Results of FTIR spectra **(A)** and thermal analysis of the three groups of pericardium. Thermogravimetric analysis showed no significant differences among the three groups. **(B)** The thermal denaturation temperature of the ADH and OHA samples was distinctly improved compared to that of the GA samples **(C)**.

The hydrazide moiety of ADH reacts with the free aldehyde group in the Glut-crosslinked pericardium to generate a stable hydrazone linkage, which neutralizes the aldehyde group. Neutralization of ADH can retain reactive primary amino groups in tissues and provide reaction sites for OHA loading. The FTIR spectra of the three groups of pericardium confirmed these reactions. As shown in Figure 5A, compared with the GA group, the absorption at 1,665 cm^−1^ in the ADH specturm was evidently decreased, indicating that ADH cross-linking consumed carbon oxygen double bonds of aldehyde or carboxyl groups. Moreover, a new absorption peak appeared at 1,630 cm^−1^, indicating that the primary amino groups remained. Furthermore, compared with the ADH spectrum, the peak at 1,665 cm^−1^ in the OHA spectrum evidently increased while that at 1,630 cm^−1^ was reduced, indicating that OHA loading consumed primary amino groups and increased the number of carbon-oxygen double bonds in carboxyl groups. These results demonstrated that ADH reacts with the aldehyde groups, forming adipic acid dihydrazone linkers.

TGA and DSC were utilized to evaluate the thermodynamic changes of ADH neutralization and OHA loading on Glut-crosslinked pericardium. TGA analysis revealed no differences in the thermal degradation of the pericardial samples of the GA, ADH and OHA pericardial samples (Figure 5B). In addition, the thermal denaturation temperature of collagen, a key factor related to collagen stability, was identified by DSC analysis. The results showed that the thermal denaturation temperatures in the ADH group (88.5°C ± 0.3°C) and HA group (89.4°C ± 0.5°C) were significantly higher than that in the GA group (86.9°C ± 0.4°C) (Figure 5C). These results indicated that neutralizing of free aldehyde groups and loading of OHA did not decrease the thermal properties of the Glut crosslinked bovine pericardium.

### 3.6 Cytocompatibility performance *in vitro*

**FIGURE 6:**
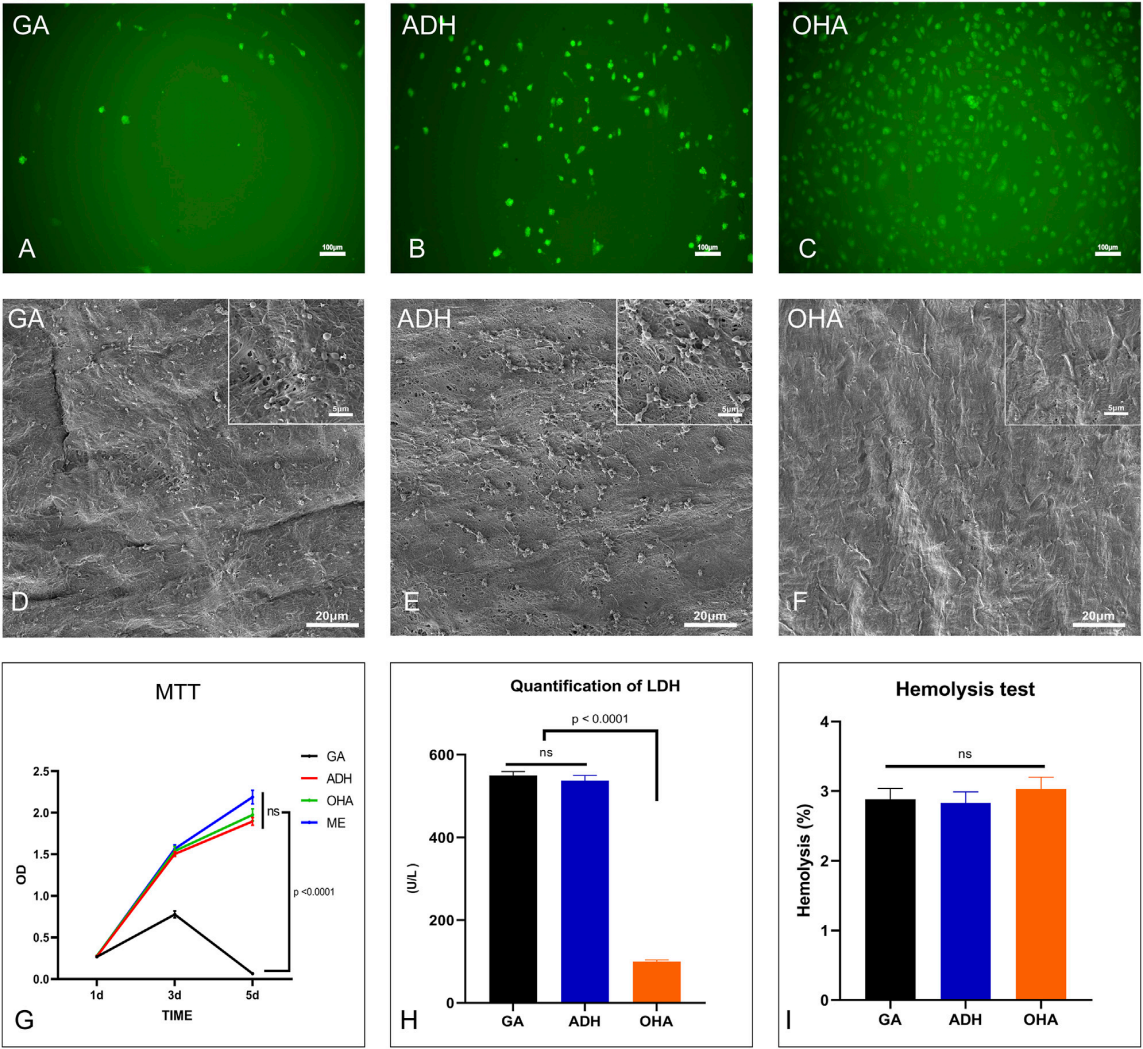
The cytocompatibility and hemocompatibility of the three groups. Human umbilical vein endothelial cell (HUVEC) proliferation and morphology were assessed by 6-CDCFDA live cell fluorescent staining. HUVECs almost completely covered the surfaces in the OHA group **(C)** on day 5, while only a few cells were present on the surfaces in the GA and ADH groups **(A, B)**. The cytotoxicity of the leaflets was determined by MTT assay, and the relative growth ratios of the GA group were significantly lower than those of the ADH and OHA groups **(G)**. Platelet adhesion on the surface of the samples was evaluated by SEM imaging **(D–F)** and LDH assay **(H)**. LDH levels in the OHA group were significantly lower than those in the GA and ADH groups. There was no significant difference in the hemolysis rate among the three groups **(I)**.

HUVECs were used to assess the toxicity and viability of the GA, ADH and OHA samples. Utilizing live cell 6-CDCFDA fluorescent staining, the proliferation and viability of HUVECs in the GA, ADH and OHA groups were observed. After 5 days of culture, the cell number on the surface of the OHA samples increased over time, whereas the number of cells on the ADH and GA surfaces did not increase. The surface of the OHA group was almost completely covered by HUVECs on day 5, while only a few cells could be observed on the surface in the GA and ADH groups (Figures 6A–C). These results suggested that loading OHA could promote the adhesion and proliferation of endothelial cells.

Cytotoxicity was then evaluated by the MTT test. After 1, 3, and 5 days of culture, RGRs of the cells cultured in pericardial leach fluid were assessed, as shown in Figure 6G. On day 1, there were no evident differences between the four groups. However, the RGRs of the cells in the GA group were significantly lower than those in the ADH and OHA groups on day 3 and day 5. Additionally, the cells in the GA group were mostly non-viable on day 5, which indicated that the ADH and OHA samples had low cytotoxicity.

### 3.7 Hemocompatibility performance *in vitro*

The hemocompatibility of the GA, ADH, and OHA samples was evaluated by hemolysis rate, platelet adhesion and LDH activity. Platelet adhesion was assessed by SEM and LDH assays. The SEM images showing platelet adhesion demonstrated that the surfaces of the GA and ADH samples were covered by a large number of spreading platelets (Figures 6D, E). In contrast, a small number of non-activated platelets were observed on the surface of the OHA samples (Figure 6F). Furthermore, the LDH content of the pericardial surface was determined, and the LDH content in the OHA group was significantly lower than that in the NA and GA groups (Figure 6H), which indicated the number of platelets adhered to the pericardial surface. In addition, the hemolysis test of the samples showed that the hemolysis rate of all three groups was <3% (Figure 6I), which meets the safety criteria for blood-contacting materials (<5%) (Totea et al., 2014).

### 3.8 Characteristics of neointima *in vivo*

**FIGURE 7:**
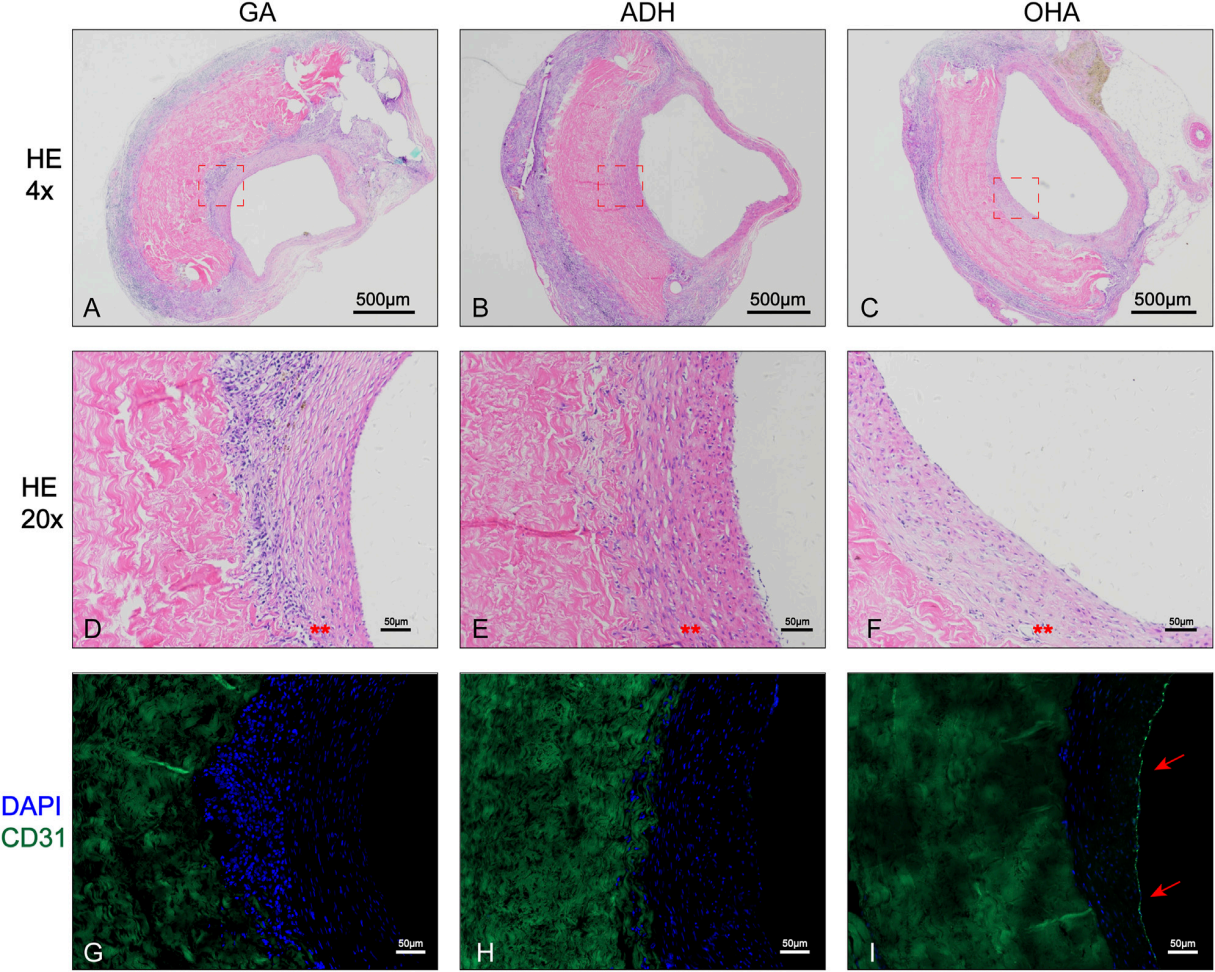
Histological analysis of the rat infrarenal abdominal aorta patch repair model. Images show the 4x **(A–C)** and 20x **(D–F)** results of HE staining in the three groups, and typical cobblestone-like cells could be observed in the OHA group . Immunofluorescence staining for the endothelial cell marker CD31 **(G–I)** showed complete CD31^+^ cell coverage in the OHA group.

**FIGURE 8:**
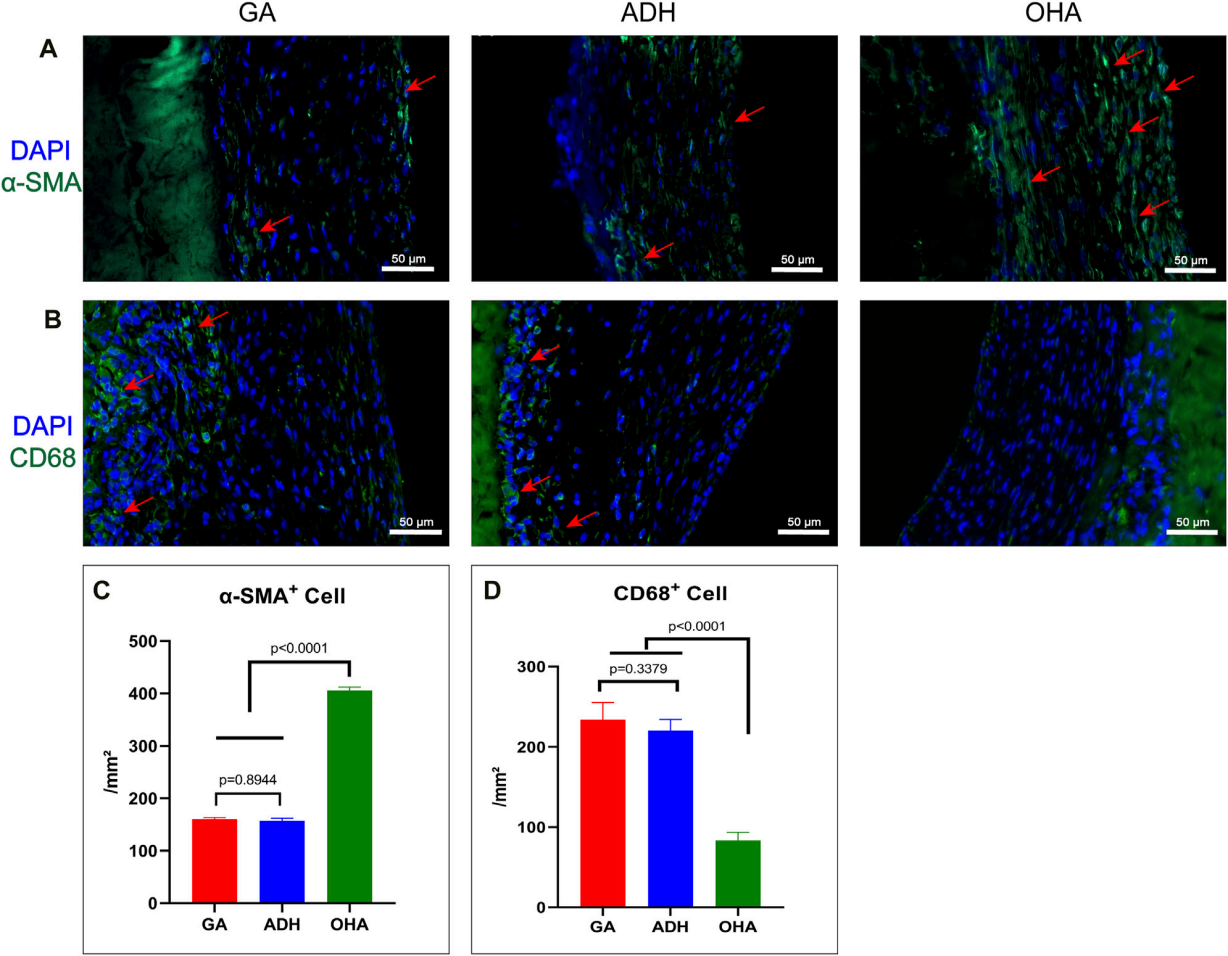
Immunofluorescence staining with α-SMA^+^ antibody and CD68 antibody was performed to observe the infiltration of smooth muscle cells and macrophages in the neointima of the GA, ADH and OHA groups, and the corresponding quantitative analysis was performed at 4 weeks. Representative images of immunofluorescence staining with anti-α-SMA (green) and CD68 (green) antibodies in each group at 4 weeks. Nuclei were counterstained with DAPI. Scale bar, 50 μm **(A, B)**. The number of α-SMA^+^ and CD68^+^ cells in the neointima was quantified at 4 weeks **(C, D)**. Red arrows indicate the locations of positive cells.

The rat infrarenal abdominal aortic patch repair model was employed to further evaluate the endothelialization performance of the types of pericardium *in vivo*. After 28 days of implantation, all animals survived without complications. Additionally, the pericardial patch repaired vessels were harvested and subjected to histological and immunofluorescence staining. Grossly, occlusion was observed in each the ADH (*n* = 1) and GA (*n* = 1) groups, while the patency was 100% in the ADH group. The results of HE staining revealed that there was more cellular infiltration in the neointima in the GA group (Figures 7A, D) and ADH group (Figures 7B, E), but the distribution was not uniform, and no classical cobblestone-like cells were observed on the luminal surface of the vascular patch. In contrast, the neointimal structures resembled native vascular structures with a uniform cellular distribution and typical cobblestone-like cells were observed on the luminal surface of the vascular patch in the OHA group (Figures 7C, F). The endothelialization of the pericardial patches was further verified by immunofluorescent staining for the endothelial cell marker CD31. In the OHA group, CD31^+^ cells covered almost the entire vascular patch (Figure 7I), while complete coverage of CD31^+^ cells was not observed on the luminal surface of the vascular patch in the GA and ADH groups (Figures 7G, H). These results were consistent with the *in vitro* cytocompatibility performance data. In addition, the infiltration of α-SMA^+^ and CD68^+^ cells was assessed to analyze a more detailed characterization of the neointima. In the OHA group, a layer of uniformly distributed of α-SMA^+^ cells were observed in the neointima, and the number was significantly higher than that in the GA and ADH groups (Figures 8A, C). In contrast, few CD68^+^ cells were observed in the neointima, and the number was significantly lower than that in the GA and ADH groups (Figures 8B, D). These results showed that the OHA group has better performance *in vivo*.

### 3.9 Anti-calcification and immunocompatibility performance *in vivo*

**FIGURE 9:**
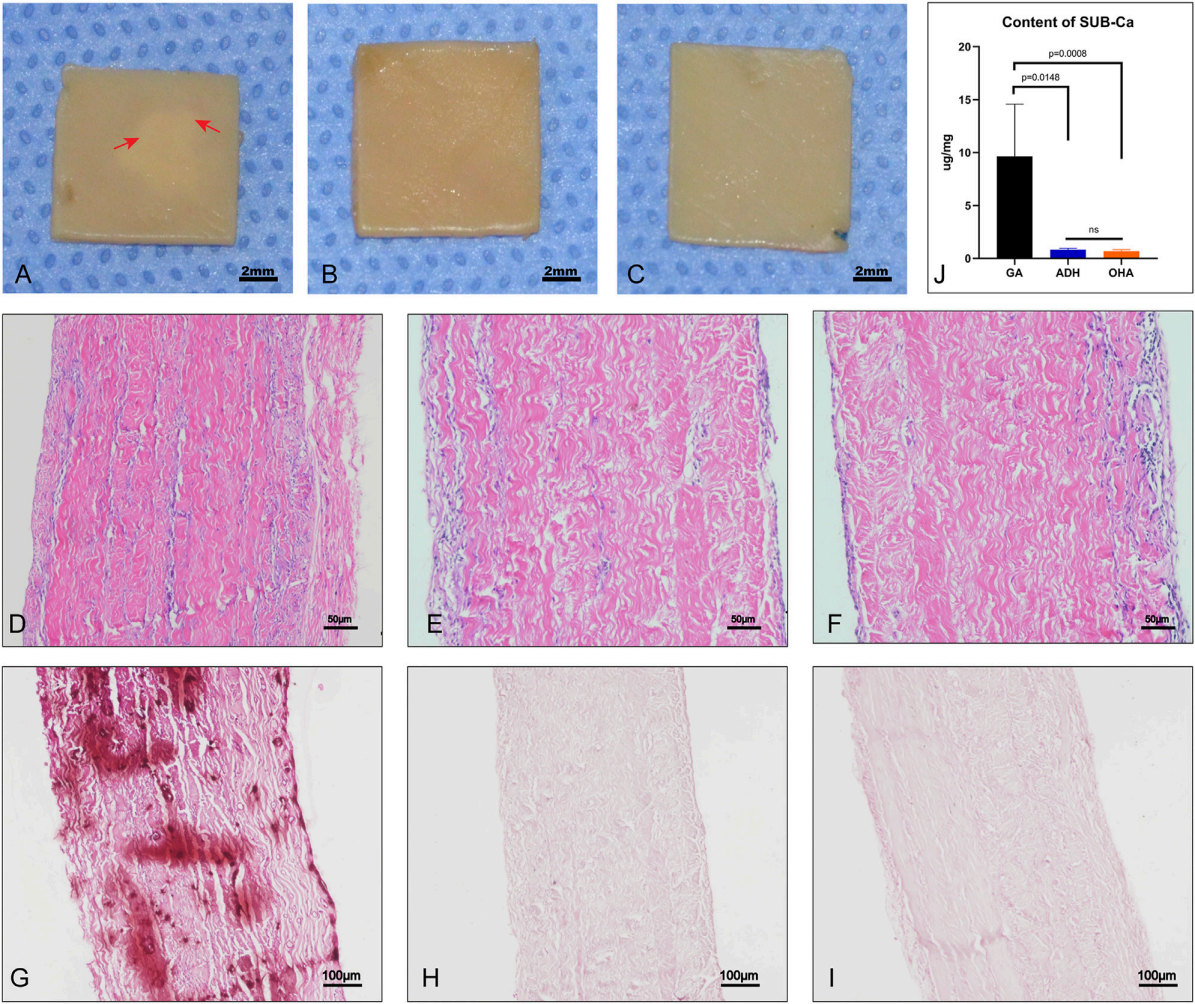
Gross specimens, histological characteristics and quantitative analysis after subcutaneous implantation for 60 days. Significant calcifications could be observed in the pericardium of the GA group **(A)**, whereas no calcifications were observed in the ADH and OHA groups **(B, C)**. The images show HE staining **(D–F)**. Alizarin red staining showed that the GA group **(G)** had more calcium deposition (dark red) than the ADH and OHA groups **(H, I)**. The calcium quantitative analysis showed that the calcium content of the ADH and OHA groups was significantly lower than that of the GA group **(J)**.

Pericardial sheets from each group were implanted subcutaneously for 60 days to evaluate the anti-calcification performance *in vivo*. Grossly, typical calcification sites were observed on part of the pericardium in the GA group (Figure 9A), but not in the ADH and OHA groups (Figures 9B, C). HE staining and Alizarin red staining were then performed, and the results showed that more cellular infiltration and obvious calcium deposition (dark red) in the Glut-crosslinked tissues (Figures 9D, G). However, no obvious cellular infiltration or calcium deposition was observed in the ADH group (Figures 9E, H) or OHA group (Figures 9F, I). Quantification of the calcification was performed using ICP-OES. As shown in Figure 9J, the calcium content of the GA, ADH, and OHA groups were 9.91 ± 4.87 μg/mg, 0.93 ± 0.13 μg/mg and 0.63 ± 0.1 μg/mg of dry samples, respectively. These results indicated that the Glut-crosslinked tissues inevitably undergo calcification, while ADH and OHA treated tissues can effectively reduce tissue calcification *in vivo*.

**FIGURE 10:**
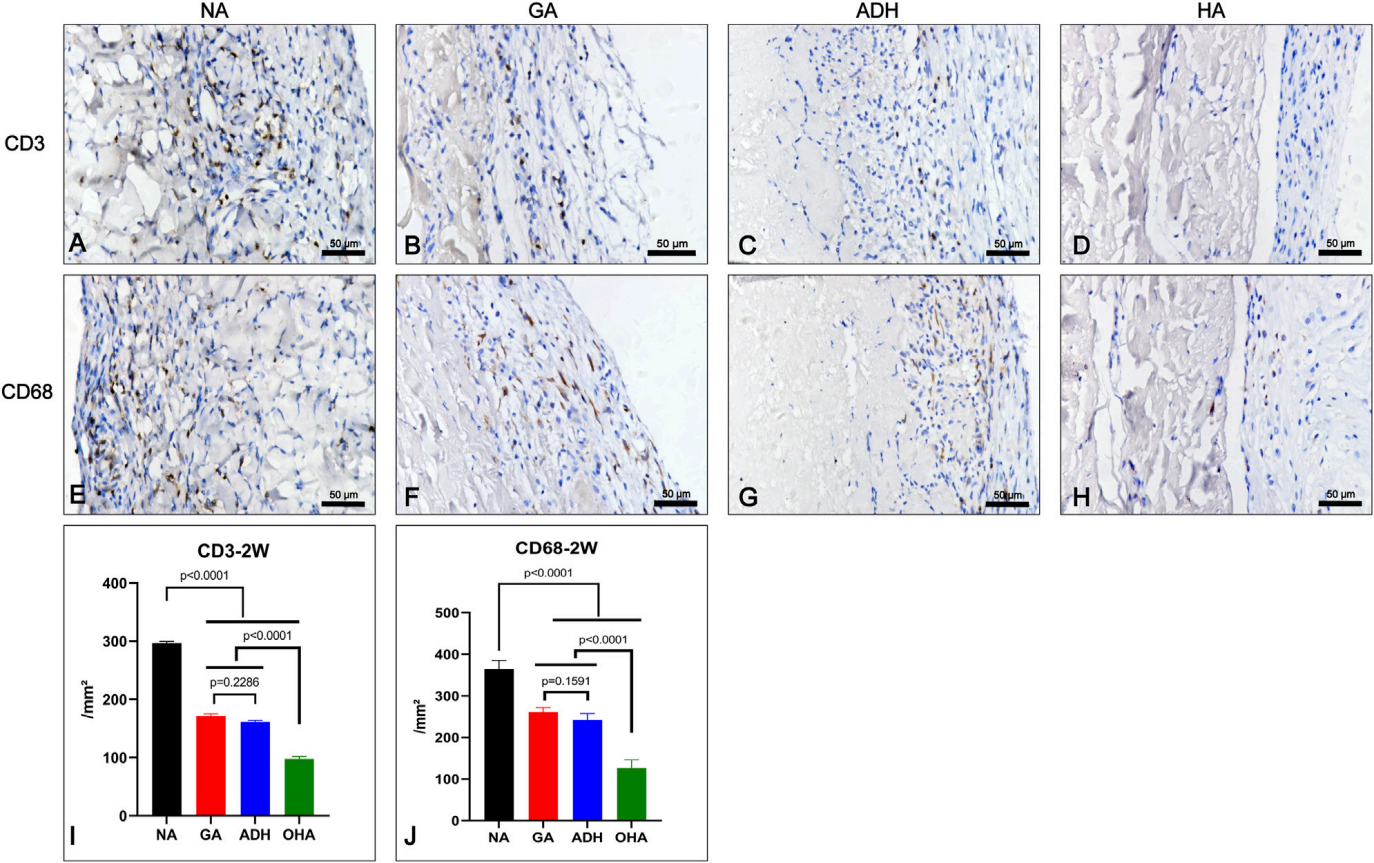
Characterization of the inflammatory response of the samples after subcutaneous implantation for 2 weeks. The images show the immunohistochemical staining of CD68 and CD3 in each group **(A–H)** and the corresponding quantitative analysis at 2 weeks **(I, J)**.

To investigate the inflammatory response of the samples *in vivo*, the infiltration of inflammatory cells was detected at 2 weeks after implantation (Figure 10). The results of IHC revealed that a large number of CD3^+^ and CD68^+^ cells were observed in the NA group (Figures 10A, E), and the number was significantly higher than that in the GA group (Figures 10B, F), ADH group (Figures 10C, G) and OHA group (Figures 10D, H), indicating that a severe immune inflammatory response was triggered *in vivo*. Notably, the OHA group had limited inflammatory cell infiltration, and the number of inflammatory cells (CD3^+^ and CD68^+^) was significantly lower than that in the NA and GA groups (Figures 10I, J), indicating that the activated inflammatory response was milder *in vivo*.

## 4 Discussion

Because the number of valve replacement surgeries is increasing, BHVs have become one of the main valve substitutes in the last decade (Manji et al., 2012). Despite the superior properties of BHVs, dysfunction and deterioration may occur due to poor endothelialization and inevitable calcification (Tam et al., 2015; Guo et al., 2021). Currently, most commercial BHV products are crosslinked with Glut, which is beneficial for biomaterial stabilization. However, several drawbacks, such as residual free aldehydes, cytotoxicity, poor biocompatibility, and calcification, are frequently encountered with Glut-crosslinked BHVs (Liu et al., 2019). Although many studies are exploring new crosslinking reagents or methods to replace Glut, none of them have been applied in the clinic (Tam et al., 2015; Hu et al., 2021; Liu et al., 2022). Neutralizing the free aldehyde groups in Glut-fixed BHVs can enhance tissue durability, reduce calcification potential, and even stimulate partial endothelialization (Gulbins et al., 2006). In addition, HA is a key component in valve tissue and is essential for maintaining the valve’s non-destructive movements and cell proliferation and differentiation (Collins and Birkinshaw, 2013; Bramsen et al., 2022). However, the level of GAGs in bovine pericardium is relatively low (Song et al., 2021). Therefore, increasing the natural HA content in the ECM is a promising strategy for the production of a new generation of BHVs.

In this study, the free aldehyde groups in Glut-crosslinked bovine pericardium were neutralized by ADH followed by OHA loading. The main advantage of this method is that it successfully neutralizes the free aldehyde groups in the tissues and provides more reactive sites for OHA loading, which increases the content of OHA in tissues by taking advantage of the bifunctional amino groups of ADH. In addition, this study is the first to demonstrate that ADH can successfully block free aldehyde groups in tissues and that unmodified natural OHA-loaded Glut-crosslinked bovine pericardial tissue can improve endothelialization and anti-calcification effects *in vivo*.

ADH is a bifunctional compound and the most suitable hydrazide crosslinker. ADH can be crosslinked with hyaluronic acid as a protein drug carrier or react with aldehyde groups to generate relatively stable hydrazone linkages, which are especially suitable for connecting glycoproteins (Li et al., 2016). Although ADH has been used in previous studies to link aldehyde groups in biomaterials (Chen et al., 2018), no studies have investigated whether ADH can completely neutralize residual aldehyde groups in Glut-crosslinked tissues and whether such treatment affects the biomechanics, thermodynamics, and biocompatibility of the material. In our study, qualitative and quantitative analyses of the free aldehyde groups in the tissues and FTIR analysis indicated that ADH could completely neutralize the residual aldehyde groups in tissues. Furthermore, evaluation of the biomechanical properties, microstructure and collagen stability of the neutralized aldehyde-based treated pericardium showed results similar to those observed with Glut-crosslinked pericardium, which indicated that the detoxification of Glut-crosslinked tissues would not cause structural changes. In terms of thermal analysis, the ADH-neutralized pericardium showed a higher thermal denaturation temperature than the Glut-crosslinked pericardium, which could be because ADH may also crosslink with other tissue components. In terms of cytocompatibility and hemocompatibility, ADH reduced the toxicity of the free aldehyde groups in tissues and cells. The evolution of calcification *in vivo* also demonstrated that reducing the tissue free aldehyde content prevented calcium deposition. These results showed that appropriate detoxification of Glut-crosslinked tissue makes it suitable for further research and potential clinical application (Meuris et al., 2018).

HA, a major type of GAG in valve tissue, plays key roles in regulating the homeostasis of heart valves (Stephens et al., 2008; Krishnamurthy et al., 2017; Lei et al., 2021), maintaining tissue hydration and flexibility, and adjusting tissue viscoelasticity. In addition, HA functions in ECM assembly, cell proliferation and migration, and tissue development (Simpson et al., 2016; Krishnamurthy et al., 2017). However, native valves or BHVs exhibit fibrotic changes in tissues due to the loss of GAGs and increased collagen expression, ultimately leading to calcification of the valve leaflets (Krishnamurthy et al., 2017). Previous studies have also demonstrated that the loss of HA in tissues is linearly correlated with calcification (Johansson et al., 2011). Our previous study showed that the GAG content in pericardial tissue was only 2%–4% in pericardial tissue, which is far lower than that in valve tissue (Song et al., 2021; Liu et al., 2022). Therefore, increasing the HA content in pericardial tissues might enhance the anticalcification properties of BHVs.

In our study, pericardial tissue was loaded with OHA after the ADH-mediated neutralization of free aldehyde groups in Glut-crosslinked pericardium, and satisfactory results were achieved in both *in vivo* and *in vitro* evaluations. In terms of OHA loading capacity, the OHA content reached 108 μg/mg in the pericardium after ADH neutralization, which was significantly higher than that in the Glut cross-linked pericardium. This increase was due to the treatment with ADH, which increased the content of primary amino groups in the tissues to improve the loading efficiency of OHA, and the results of the NHN test and FTIR analysis supported this modification. In addition, there were no significant changes in the biomechanical properties, microstructure, collagen stability or thermal characteristics of OHA-loaded pericardial tissue, which also indicated that OHA loading caused no structural damage to Glut-crosslinked valve tissue. However, OHA-loaded pericardial tissue had better hydrophilicity, suggesting that OHA may reduce thrombus formation by the material (Bui et al., 2019), and in the platelet adhesion test, OHA-loaded pericardium significantly reduced platelet adhesion, suggesting that it may have better antithrombotic properties.

Calcification is a major obstacle that limits the operation life of commercial BHVs, prompting the stiffening of the heart valves and hindering the normal opening and closing movement of the valves, ultimately leading to BHV dysfunction. The mechanism of calcification is currently unknown. The loss of HA, the presence of residual aldehyde groups, and cytotoxicity have been suggested as potential contributors to calcification (Johansson et al., 2011; Manji et al., 2012). In this study, ADH neutralization, which significantly reduced the free aldehyde groups in tissues while decreasing cytotoxicity and OHA loading and filling further occluded potential calcification sites in the tissues and improved the anticalcification properties of the material. An embedded subcutaneous rat model was used to evaluate calcification, and after 60 days of implantation, the calcium contents in the ADH and OHA groups were significantly lower than those in the GA group, indicating that the ADH and OHA samples have better anti-calcification capabilities.

HA physiological function and molecular size are related, and high-molecular-weight HA is associated with space filling, antiangiogenesis and anti-inflammation (Stern et al., 2006). Low-molecular-weight HA is associated with inflammation regulation, angiogenesis and the inhibition of endothelial mediator transformation and other processes (Rodgers et al., 2006). Heterogenic BHV can cause inflammatory reactions when implanted *in vivo*. Uncrosslinked bovine pericardium could cause a severe immune-inflammatory response that may be associated with the natural barrier. The results of the early inflammatory response of the samples *in vivo* showed that the inflammatory cells of the NA group were extensively infiltrated in the leaflet. In contrast, the OHA group had a milder immune inflammatory response than the GA, ADH and NA groups. These results indicated that the OHA may better inhibit inflammatory cell infiltration. Moreover, studies performed *in vitro* have shown that OHA can induce the migration and proliferation of endothelial cells (Montesano et al., 1996). Notably, low-molecular-weight HA stimulates the RAS/Raf/ERK1/2 and protein kinase C pathways to promote the proliferation of endothelial cells (Slevin et al., 1998). Valvular endothelial cells play an important role in valve development and disease. An intact endothelial cell layer can act as a physical barrier that prevents inflammatory responses and thrombosis (Boström et al., 2011; Jiang et al., 2016). The residual free aldehyde groups present in Glut-crosslinked biomaterials have been shown to be responsible for the inability to re-endothelialize due to their severe cytotoxicity (Guo et al., 2018). The vascular smooth muscle layer is crucial for maintaining the mechanical strength and vasoactive responsiveness of blood vessels in coordination with the endothelial layer. Previous studies have confirmed that HA can serve as a bioactive molecule to regulate smooth muscle regeneration (Gouëffic et al., 2006; Qin et al., 2020). In our study, the OHA-loaded pericardium was evaluated for endothelialization *in vitro* and *in vivo*, and both studies indicated that the pericardium in the OHA favored more for endothelial cell coverage. In the abdominal aorta patch repair model, intact CD31^+^ cell coverage on the pericardial patches was observed in the OHA group, while no intact CD31^+^ cell coverage was observed in the GA and ADH groups. Moreover, the results also demonstrate that the loading of HA markedly increased the number of the smooth muscle layers (α-SMA^+^). All these results proved that OHA improved the biocompatibility of Glut-crosslinked pericardium *in vivo*.

## 5 Conclusion

In this study, we explored an optimized method for creating Glut-crosslinked BHVs. Pericardial tissue with ADH-mediated neutralization of free aldehyde groups loaded with OHA exhibited better biocompatibility, lower cytotoxicity, better anti-inflammation and anti-calcification properties and endothelialization potential. This innovative optimized treatment would allow GLUT-crosslinked BHVS to have an improved clinical life span.

## Data Availability

The original contributions presented in the study are included in the article/supplementary material, further inquiries can be directed to the corresponding authors.
